# PAZAR: a framework for collection and dissemination of cis-regulatory sequence annotation

**DOI:** 10.1186/gb-2007-8-10-r207

**Published:** 2007-09-28

**Authors:** Elodie Portales-Casamar, Stefan Kirov, Jonathan Lim, Stuart Lithwick, Magdalena I Swanson, Amy Ticoll, Jay Snoddy, Wyeth W Wasserman

**Affiliations:** 1 Centre for Molecular Medicine and Therapeutics, CFRI, University of British Columbia, Vancouver, BC., V5Z 4H4, Canada; 2 Graduate School for Genome Science and Technology, Oak Ridge National Laboratory-University of Tennessee, Oak Ridge, TN, 37830, USA; 3 Applied Genomics Department, Pharmaceutical Research Institute, Bristol-Myers Squibb, NJ, 08534, USA; 4 Biomedical Informatics Department, Vanderbilt University School of Medicine, Nashville, TN, 37235, USA

## Abstract

PAZAR is an open-access and open-source database of transcription factor and regulatory sequence annotation with associated web interface and programming tools for data submission and extraction. Curated boutique data collections can be maintained and disseminated through the unified schema of the mall-like PAZAR repository. The Pleiades Promoter Project collection of brain-linked regulatory sequences is introduced to demonstrate the depth of annotation possible within PAZAR. PAZAR, located at , is open for business.

## Abstract 2

PAZAR is an open-access and open-source database of transcription factor and regulatory sequence annotation with associated web interface and programming tools for data submission and extraction.

## Rationale

The study of gene regulation has emerged as a focus of efforts to understand how genome sequences give rise to diverse and complex cells and tissues. From gene-centric dissection of promoter sequences [1] to regulon-based analysis of cis-regulatory modules [2] through to genome-scale chromatin probes [3], researchers across the subdisciplines of modern biology strive to understand how cells regulate the flow of genetic information from DNA to RNA via the process of transcription. This developing knowledge, and more critically the data produced, has unleashed a wealth of computational-driven approaches to predict the locations of regulatory sequences, as well as to discover classes of binding sites for transcription factors and models of regulatory programs [4-8]. Annotated sets of regulatory sequences, with well understood and independently confirmed function, are necessary to serve as gold standards to support the validation of new molecular techniques and computational algorithms. As confidence in regulatory annotation and prediction advances, researchers will increasingly draw on such knowledge to design sequences capable of directing targeted gene expression in molecular applications such as gene therapy.

Existing regulatory sequence data collections are generated primarily in a need-driven manner. A dedicated researcher pursuing an idea will extract from the scientific literature a sufficient set of annotations to support their own studies. For example, the widely used JASPAR collection of transcription factor binding profiles [9] was developed initially for the study of binding pattern similarities across families of structurally related transcription factors [10]. Similarly the ORegAnno database [11] was compiled initially for the study of genetic variations known to alter binding sites of transcription factors. The best of these reference collections are subsequently used by researchers within bioinformatics to improve and assess the performance and efficiency of computational methods. These boutique data collections are the backbone of the current generation of regulatory sequence analysis studies (examples include [9,11-20]). It is our perception that boutique reference databases will likely remain the primary sources for regulatory sequence annotations for much time to come. While large centrally curated database have emerged for proteins (UniProt [21]) or human genetics (OMIM [22]), funding for large-scale curation of an open-access regulatory sequence collection appears unlikely.

The existing pool of annotated data for transcriptional regulation is not optimal. There is an unfortunate long-term problem that stems in part from the fact that database maintenance is tiresome. The operators of the boutique databases quickly move on to other tasks, motivated equally by a dearth of monetary support and the excitement of the next project. Few regulatory sequence collections have endured for long periods of time with evidence of substantial expansion. The widely used TRANSFAC collection of regulatory sequences has been a central tool for bioinformatics [23]. However, the transfer of the collection to a commercial funding model makes it difficult for the system to build on community participation. The scientific community is less likely to add to and improve upon data annotation distributed in a for-profit tool. Limited commercial curation may tend to focus on commercially relevant annotation rather than basic science research needs.

The boutique model of database development suffers from several fundamental problems. As mentioned, collections can stagnate after the initial enthusiasm of the creator wanes. For current research, reference collections must increasingly map onto genome sequence coordinates, and thus the utility of the collections rapidly diminishes if such coordinates are not kept up to date. Furthermore, data need to be delivered in a dynamic manner accessible by web interfaces, programming interfaces and emergently via support of semantic interfaces. Flat file data models are too rigid and cannot capture data at its full granularity.

**Figure 1:**
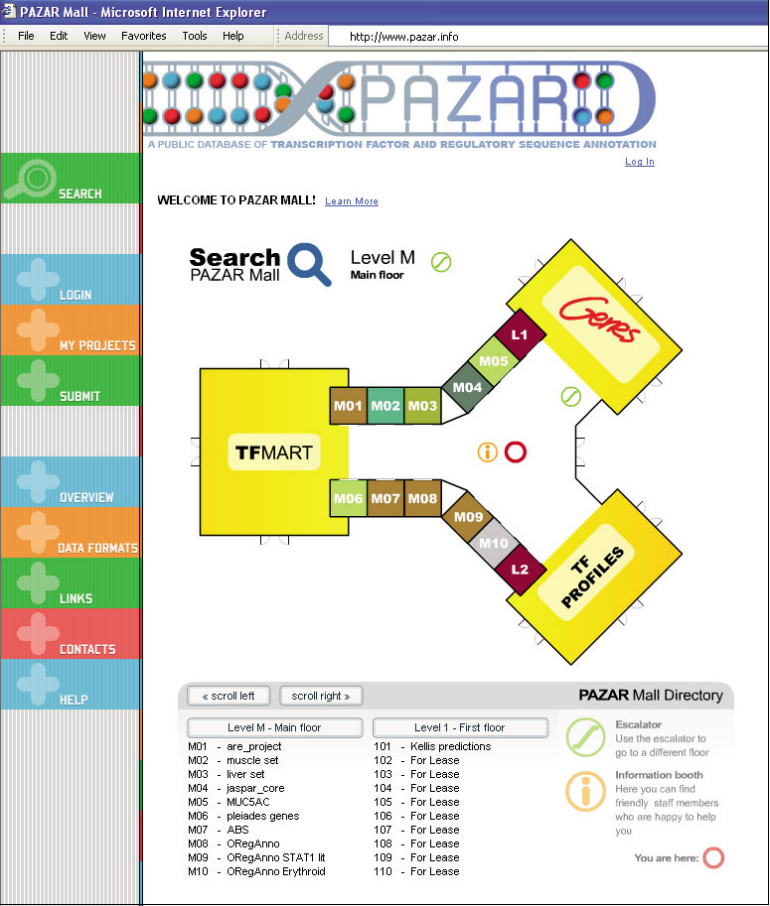
PAZAR Mall. The PAZAR database can be viewed as a mall bringing together independent boutiques. The user can visit each store separately by clicking on the corresponding boutique and search through the data using various filters. Global search engines, allowing searching of the entire PAZAR mall, are available by clicking on one of the three department stores. The user can then search PAZAR by gene (Genes), transcription factor (TFMART), or transcription factor binding profiles (TF PROFILES).

In this report we introduce the PAZAR information mall for regulatory sequence annotation (Figure 1). Building on the resource of boutique database owner-operators, PAZAR (the Bulgarian word for shopping mall) provides a computing infrastructure for the creation, maintenance and dissemination of regulatory sequence annotation. Incumbent upon the purpose, PAZAR provides tools for data exchange (XML and GFF formats), dynamic data access (application programming interface) and internet-based user interaction. In order to provide a framework for independent data boutiques, PAZAR utilizes an extremely flexible data schema to support a broad range of data annotation. While PAZAR itself is an open-access and open-development project, the system allows for boutique operators to limit access to a data collection in order to facilitate their ongoing collaborative projects or early stage development of reference collections. PAZAR [24] is now open for business.

## Database organization and controlled vocabularies

**Figure 2:**
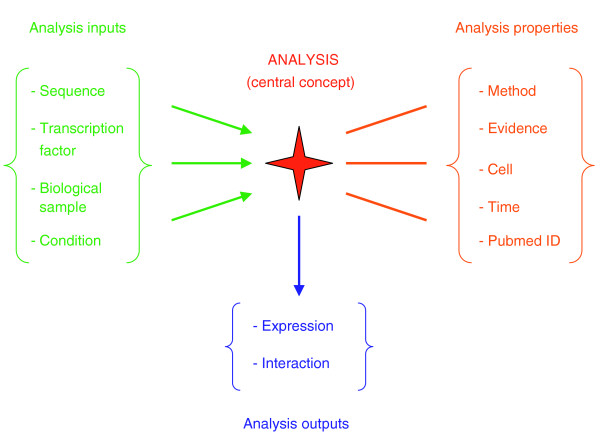
PAZAR central concept: analysis and input/output system. The sequences and transcription factors are stored independently in the database and are then linked together as inputs of an analysis. Other types of input can be used, such as a biological sample (for example, nuclear extract) or a condition (for example, addition of a chemical compound). The analysis is defined by various properties (the method and cell type used, the PubMed identifier, and so on) and links inputs and outputs together. An output could be the observed effect, for example expression response or interaction level. The system is very flexible, allowing various combinations of inputs and outputs.

PAZAR is designed around two main concepts: first, the necessity for unambiguous identification of the chromosome location for any given cis-regulatory element (CRE) using genomic coordinates (this restricts the allowed species to those for which a genome assembly is resolved); and second, a flexible database schema allowing for the capture of annotations derived from a wide range of experiments (Figure 2). In brief, nucleotide sequence and transcription factor (TF) information is stored independently. Relationships (for example, TF 1 binding to sequence A) are established through an 'analysis' object, which describes the analysis properties (the method used, the cell type in which the experiment was performed, the PubMed abstract identifier, and so on). The TF and sequence are then treated as inputs of this analysis, the output being the effect that is observed (interaction or change in expression). This representation of data gives the database significant flexibility regarding the type of information that can be captured, a characteristic that is essential for handling the diversity of annotations most often used to describe gene regulation.

This flexible design enables PAZAR to represent data consistent with our current understanding of transcriptional regulation. First, the system refers to 'transcription start region' instead of 'transcription start site' as increasing evidence shows that transcription start sites are more 'fuzzy' than previously thought and often cannot be confined to unique nucleotides [25,26]. Second, it takes into account the fact that TFs often act as complexes containing more than one subunit. For instance, members of the bZIP family of TFs, including Fos, Jun, Maf/Nrl, CREB/ATF and CEBP/NFIL-6, display subtle differences in DNA binding specificity depending on the dimers formed [27]. PAZAR is the first system to acknowledge this fact and to allow the annotator to differentiate between different dimer compositions. Furthermore, PAZAR is the first database to capture mutation data in an efficient way, enabling the user to correlate each base pair change with a change in regulatory sequence activity. We anticipate that this 'negative' information will allow for the development of more diverse TF binding models. PAZAR not only captures information on individual TF binding sites but also on the longer cis-regulatory modules at which TFs interact. In addition, to better represent data, the PAZAR system allows for the storage of TF binding profiles in matrix format. This is important in order to accommodate external data that do not provide individual binding site information, such as JASPAR [9] or computational motif predictions [28].

The aforementioned design features have been implemented using the mySQL relational database. The current database structure is developed and maintained through the DBDesigner software application, which provides an integrated graphic development interface and tools for automatic SQL script generation and data exchange.

The wide array of PAZAR hostable datasets contains a great heterogeneity of information. To overcome the challenges imposed by such data diversity, we incorporate controlled vocabularies as a means to consistently annotate regulatory sequences and expression patterns. Bio-ontologies offer common semantics for biological functional annotations [29]. Two topics requiring controlled vocabularies in PAZAR are: cell types and tissues; and experimental methods. For the former, we chose the BRENDA Tissue Ontology as our reference [30] and are providing updates to the BRENDA developers on a periodic basis as PAZAR users expand the vocabulary. With respect to the experiment type ontology, we are collaboratively working with the developers of the ORegAnno database [11].

## PAZAR web interface and programming tools

As illustrated in Figure 1, the PAZAR database can be viewed as a mall bringing together independent boutiques. The CGI-based interface builds on this theme through the incorporation of a mall map that serves as the entry to the search interface. Users can search by gene ('Genes' department store), TF ('TFMART' department store) or TF binding profile ('TF PROFILES' department store). If interested in only one specific dataset hosted in PAZAR, users can also search this specific store by clicking either on the store on the map or on its name in the mall directory.

### Use-case number 1

**Figure 3:**
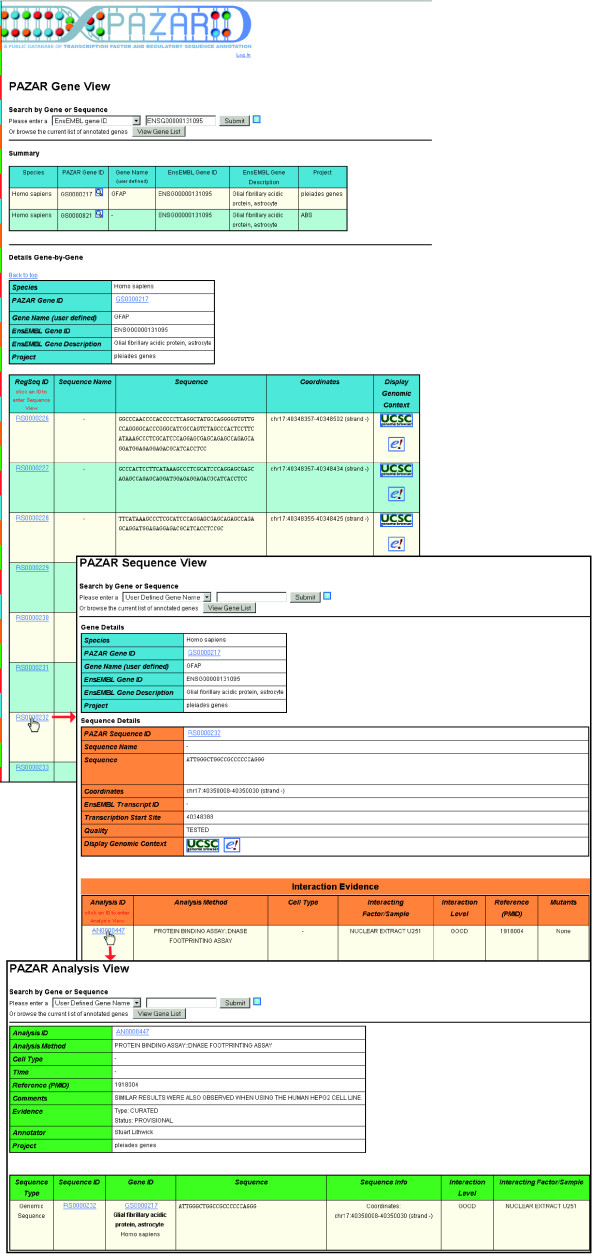
Example query: search by gene. By clicking on the 'Genes' department store at the upper right corner of the mall, users can perform a gene-specific query. One can view the list of all genes in PAZAR by clicking on the 'View Gene List' button. Alternatively, users can search for a specific gene within all of PAZAR based upon several gene-specific identifiers. At the top of the 'Gene View' page is a summary table of all of the genes obtained from the search. Here, the results show that the queried gene (EnsEMBL gene ID ENSG00000131095) has annotations in two different projects. Below, users can find the details and all annotated regulatory sequences for each of the resulting genes individually as, in PAZAR, each boutique stays independent within the mall. By clicking on the regulatory sequence ID for a specific regulatory sequence, found in the far left column, users can access the PAZAR Sequence view for that sequence. In this view, data are color-coded, with gene-specific information presented in blue and sequence-specific data in orange. A gene-specific summary table is presented at the top of the page followed by a table detailing the regulatory sequence of interest. A third table summarizes the supporting experimental data for this regulatory sequence. Clicking on the Analysis ID found in the leftmost column of this table takes users to the PAZAR Analysis View, color-coded in green and containing a more in-depth description of the supporting experimental data.

If one is looking for regulatory information for a specific gene, one should click on the 'Genes' department store and enter the gene identifier (several options are available). As a result, the gene view page is loaded with a summary table of all genes corresponding to the query. For each of the displayed genes, the list of all annotated regulatory sequences is located in tables further down the page (Figure 3). More information can then be obtained by clicking on the 'RegSeq ID' to enter the 'Sequence View' (Figure 3). From these pages one can access greater detail by clicking on the 'Analysis ID' to enter the 'Analysis View' (Figure 3). In the gene and sequence views, one can click on the UCSC or EnsEMBL icons to display the sequences within the UCSC or the EnsEMBL genome browser, respectively.

### Use-case number 2

**Figure 4:**
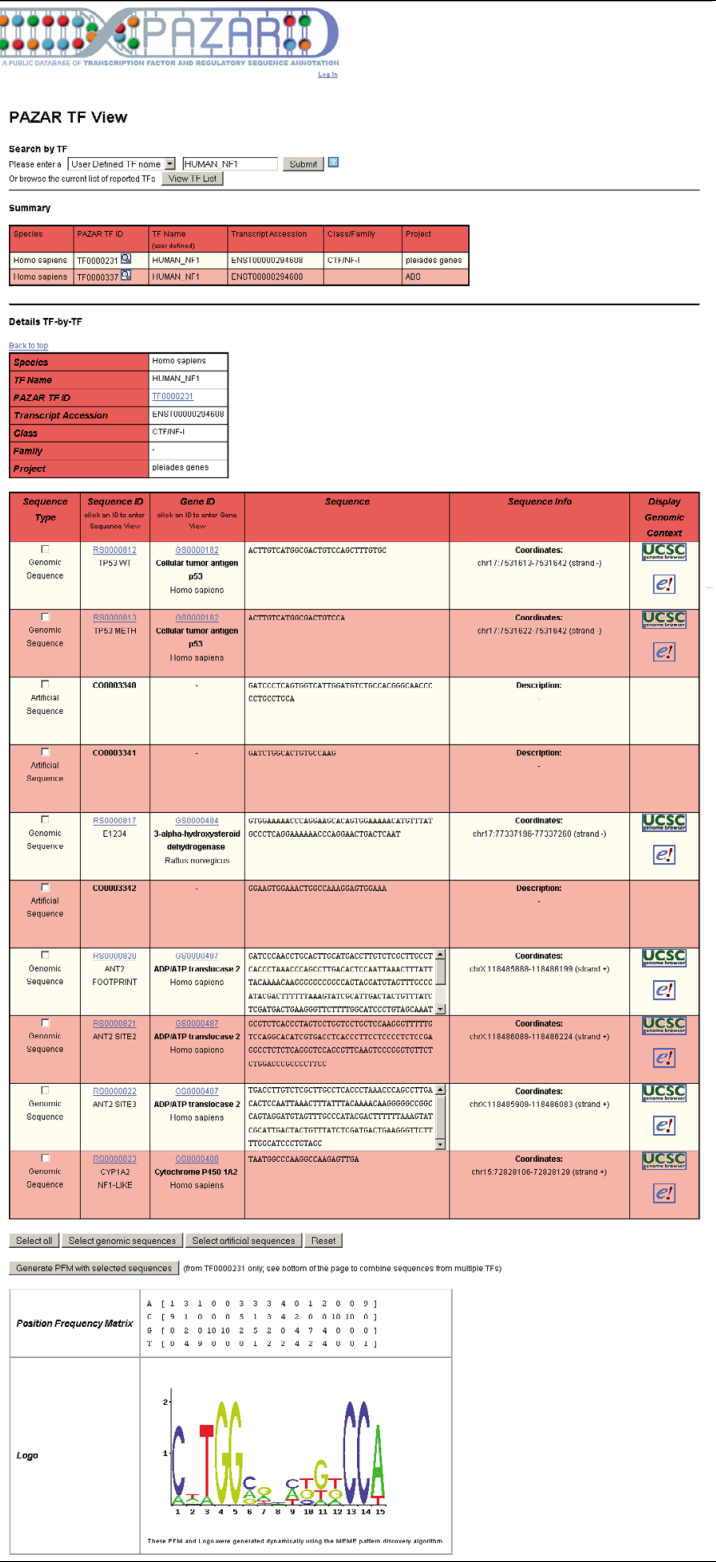
Example query: search by transcription factor. By clicking on the 'TFMART' department store at the left hand side of the mall, users can perform a TF-specific query. The 'TF View', color-coded in red, is very similar to the 'Gene View' (see Figure 3) with a summary table of all of the TFs obtained from the search at the top followed by details and binding sites for each of them individually. Here, the results show that the queried TF (HUMAN_NF1) has annotations in two different projects. The binding sites can be genomic sequences with defined coordinates or they can be artificial (for example, oligonucleotide representing a consensus sequence). All the sites are aligned and a TF binding profile is built dynamically using the MEME pattern discovery algorithm [31]. Users can construct a custom scoring matrix and binding profile based upon a subset of the sequences for that TF by clicking in the check boxes of those sequences meant to be included and clicking 'Generate PFM with selected sequences'. Alternatively, users can generate scoring matrices and binding profiles based upon just genomic or artificial sequences by clicking on 'Select genomic sequences' or 'Select artificial sequences', respectively.

When looking for binding sites for a given TF, one can use the 'TFMART' department store. Various identifiers can be used for the query and the results will be displayed in the 'TF View' (Figure 4). First, a summary table shows all available TFs corresponding to the query. Then, for each, the list of all annotated binding sites is displayed. The binding sites can refer to specific genomic coordinates, with accompanying hyperlinks that take the user to the corresponding Sequence or Gene View, or they can be artificial (for example, oligonucleotide representing a consensus sequence). All the sites are aligned and a TF binding profile is built dynamically using the MEME pattern discovery algorithm [31].

### Use-case number 3

**Figure 5:**
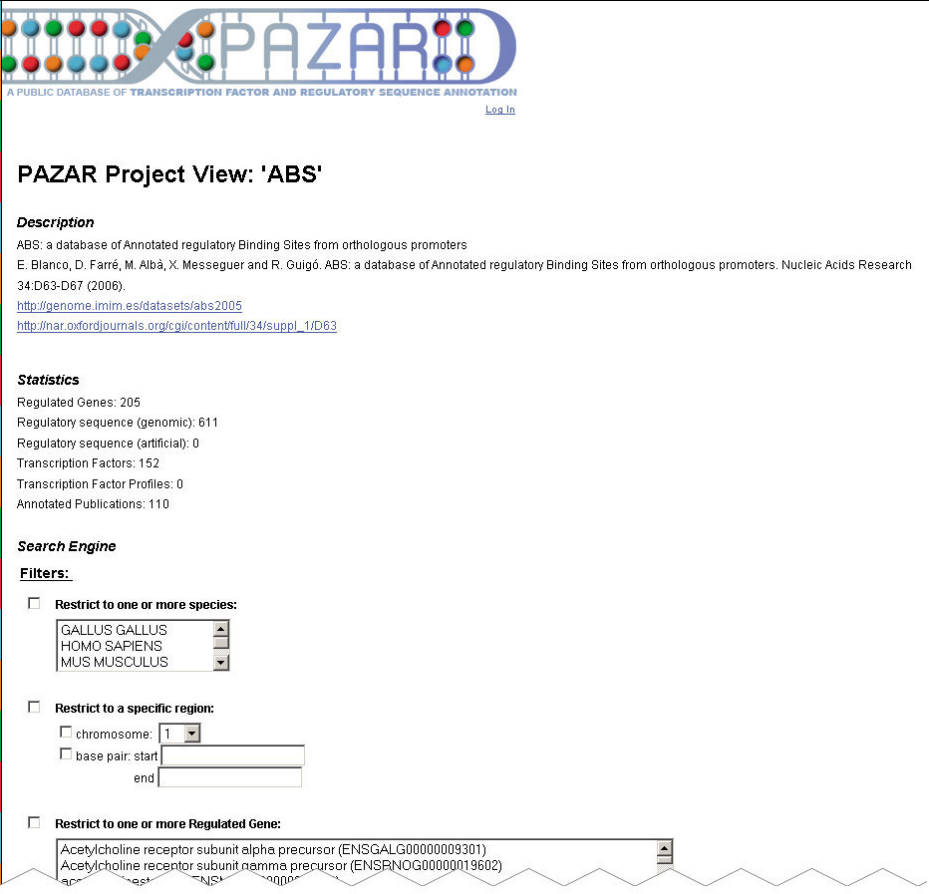
Example query: search within a specific boutique project. One might desire to limit queries to a single collection. To do so, the user must find the corresponding boutique in the mall map or directory and click on it. The 'Project View' provides a brief description of the dataset (here the ABS project) as well as some statistics on the data it contains. Below, the user can choose amongst various filters to search through the data and display it in the 'Gene View', where regulatory sequences will be grouped by the genes they regulate, or in the 'TF View', where the sequences are grouped by the TFs that bind to them.

One might desire to limit queries to a single collection. To do so, the user must find the corresponding boutique in the mall map or directory and click on it. The 'Project View' provides a brief description of the dataset as well as some statistics on the data it contains (Figure 5). Below, the user can choose amongst various filters to search through the data and display it in the 'Gene View', where regulatory sequences will be grouped by the genes they regulate, or in the 'TF View', where the sequences will be grouped by the TFs with which they interact.

PAZAR provides a submission interface that one can access by clicking on 'Submit' in the left menu. This web-based streamlined user interface provides a simplified entry point to the database for non-professional curators, such as scientists that want to deposit their own experimental data to the public repository.

We have developed a Perl API (application programming interface) that hides the intrinsic complexity of the schema from database users. The object-oriented approach provides programmers with different layers of abstraction, allowing advanced users to create 'high-layer' objects and methods to suit project-specific needs.

To best serve users, PAZAR must frequently retrieve data from external sources. For example, sequence coordinates must be updated when genome assemblies are released, updated, or re-annotated. The API pazar::talk modules make this possible by delegating all external queries to an appropriate pazar::talk::*database *module. Currently, three modules have been developed to interact with the GeneKeyDB [32], JASPAR [9], and EnsEMBL [33] databases. The open source nature of this project allows users to develop or adapt additional modules to work with any database of their choice.

A PAZAR-specific exchange format has been implemented in XML (extensible markup language). In addition to facilitating data transfer between 'boutiques' and the central master database, the XML format can support custom stand-alone user interfaces that do not have direct database access. Some basic sequence features can also be exported in GFF (general feature format). API methods are available to parse PAZAR XML or GFF format data for importation into the database.

## Database content

Each data collection within PAZAR is called a project and is identified by a project ID, a project name, a status and a list of users. The project status can be 'restricted' (only the project-specific users have read and write access), 'published' (only the project-specific users have write privileges, but everyone has read access) or 'open' (everyone has read and write privileges). For this purpose, each record in the database is linked to a project ID, allowing all projects to share the same tables within the database schema, yet retaining their project identity so that they remain independent data collections.

At the time of submission of this manuscript, there were 11 projects present in the database (Table 1). Included are the JASPAR database for TF binding profiles (core sub-database) [9], the ABS collection of annotated regulatory binding sites [14], extensively annotated genes from the Pleiades Promoter Project (see below), muscle-specific and liver-specific collections of regulatory regions [7,8], a collection of antioxidant response elements, a dataset related to the regulation of the MUC5AC gene and a collection of predicted regulatory motifs from human promoters and 3' untranslated regions [28]. We are currently in the process of importing the ORegAnno database [11]. The ORegAnno boutique within PAZAR includes the annotations directly submitted to the ORegAnno system. Externally generated collections available from the ORegAnno database are given unique PAZAR project identifiers as they are successfully imported. To date, these collections include the PennState erythroid cis-regulatory modules [34] and the ChIP-TS STAT1 literature-derived binding sites [35].

**Table 1:** PAZAR database content on 13 July 2007*

Project	Regulated genes	Regulatory sequence (genomic)	Regulatory sequence (artificial)	Transcription factors	Transcription factor profiles	Annotated publications
ABS	205	611	-	152	-	110
ARE project	14	14	-	2	-	15
JASPAR core	-	-	3,229	84	123	94
Liver set	14	62	-	-	-	-
MUC5AC	2	23	-	13	-	9
Muscle set	15	49	-	-	-	-
ORegAnno	256	690	-	115	-	305
ORegAnno Erythroid	8	33	-	1	-	1
ORegAnno STAT1 lit	28	37	-	1	-	29
Pleiades genes	206	810	95	177	-	409

TOTAL	**748**	**2,329**	**3,324**	**545**	**123**	**972**

*This table includes only the experimentally validated annotations available in PAZAR and, therefore, excludes the Kellis predictions.

**Figure 6:**
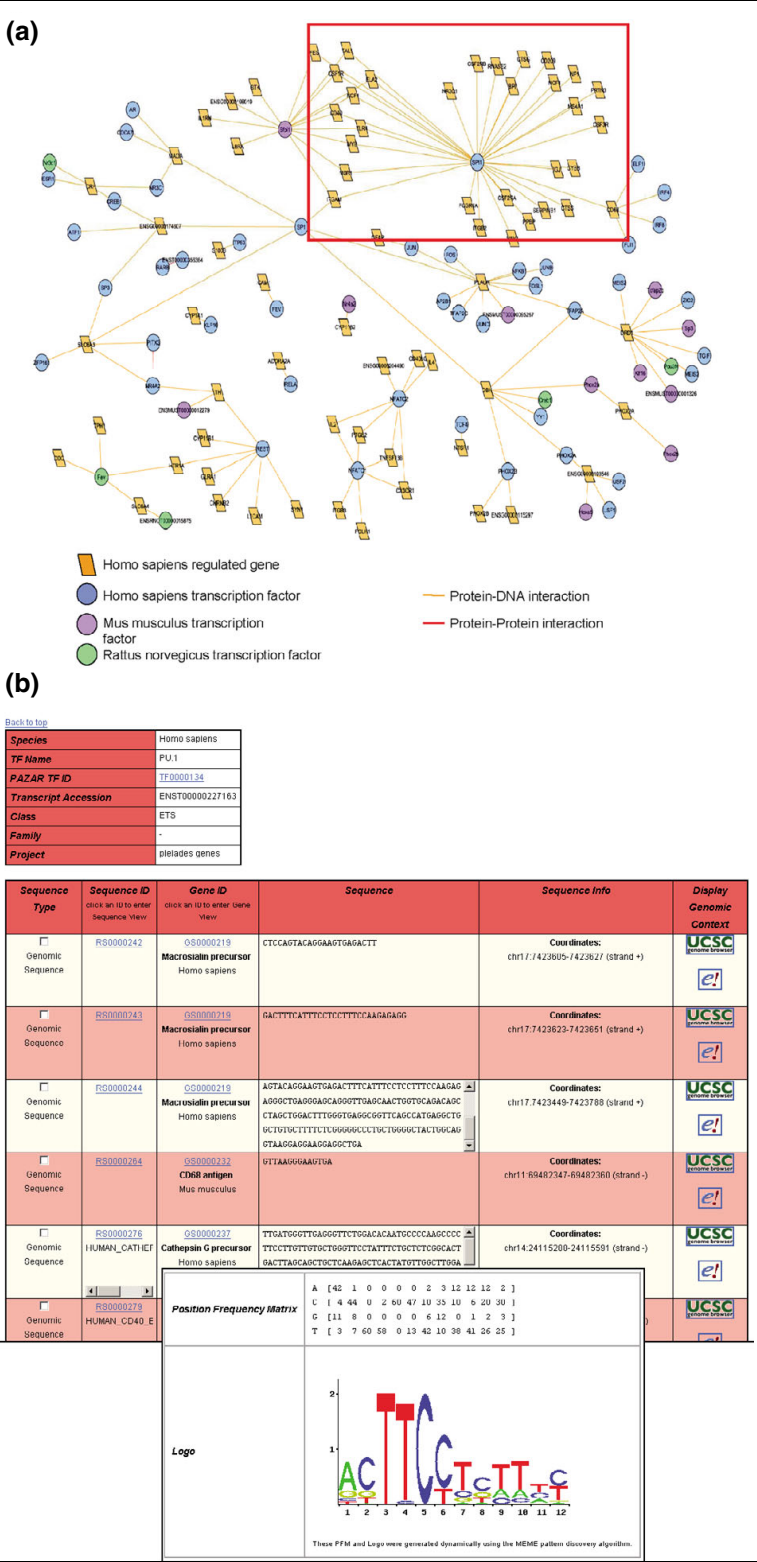
Visual representation of the human gene annotations of the 'Pleiades genes' project in PAZAR. **(a) **Cytoscape visualization. Human genes are represented as orange squares and transcription factors regulating them as circles (blue for human, purple for mouse and green for rat). The different species of transcription factors reflects the fact that assays on the regulation of human genes are often carried out in cell lines or with recombinant transcription factors from different organisms. The orange edges represent the annotated interactions between transcription factors and genes. The red edge visualizes an interaction between two transcription factors. The red box highlights the human transcription factor SPI1 (also called PU.1) and all the genes recorded as containing a transcription factor binding site for it. **(b) **PAZAR TF View detail for PU.1 annotations from the 'Pleiades genes' project. Only the first 6 binding sites (out of 60) are displayed, as well as the binding profile for the combined set dynamically generated by the MEME software [31].

The 'Pleiades genes' project is a good example of the level of annotation that can be captured in PAZAR. This dataset is being collected by data curators working for the Pleiades Promoter Project [36], a Genome Canada project focused on the creation of short regulatory sequences to drive gene expression in defined brain regions of therapeutic interest. Thus, one major component of the project is to identify genes expressed in specific brain regions and annotate their known regulatory sequences. PAZAR is used for this regulatory data collection, providing the required level of annotation details (experiments, cell types, level of interaction or expression, mutations, and so on). As an example of how data from PAZAR can be visualized, Figure 6a shows a graphical representation in Cytoscape [37] of the 'Pleiades genes' project, focusing on the human gene-TF interactions. The box in Figure 6a highlights the human PU.1 transcription factor (also known as SPI1) and all the genes containing a recorded PU.1 binding site within the 'Pleiades genes' project. Figure 6b shows the PAZAR display for those PU.1 transcription factor binding sites and the binding profile for the combined set.

## PAZAR availability and distribution

PAZAR is open-access and open-source, providing a completely transparent development and data compilation. Both the code and the data (except for any restricted projects) are available through the PAZAR website [24] or the development website [38].

## Conclusion: growth and development

A large fraction of gene regulation data comes from high-throughput techniques such as gene expression and chromatin immunoprecipitation microarrays. Unfortunately, the observed data are difficult to interpret as they often reflect contributions from overlapping processes. One means to improve the interpretation of results is to incorporate prior knowledge of regulatory processes [39,40]. The JASPAR database of TF binding profiles is widely used for such purposes [9], yet provides merely a fraction of the information necessary to support the research community. An excellent and extensive comparison of the existing binding site prediction tools [41] suggests that one of the biggest hurdles in evaluating these tools objectively is the lack of an adequate reference collection. Thus, access to a larger pool of experimentally derived reference data, such as provided by PAZAR, could facilitate both improved interpretation of high-throughput data and assessment of computational methods.

Considering the future of gene regulation databases, three things are apparent. First, the motivation and expertise of individual researchers, as well as their focus on deep annotation of specific pathways and processes, make boutique operators a key resource in long-term compilation of regulatory sequences and annotations. Second, based on principles shared by the authors, any database should provide data and software in an open, unrestricted manner to all researchers in all settings. Third, the ongoing technical challenges for databases require a long-term commitment of talented technical staff. PAZAR was developed based on these observations.

While our laboratory will maintain PAZAR for the long-term as it is necessary for our on-going research, ideally the project would expand through the engagement of a cooperative research community. Recent events suggest that the global research community is prepared to participate in regulatory sequence annotation projects. In late 2006, a group of open-access motivated scientists contributed regulatory sequence annotations to the ORegAnno database [11]. While PAZAR and ORegAnno differ substantially in mission and approach, both address the need for open-access data collections and the developers are working together on common components such as controlled vocabularies. Contributions to a shared system could be combined synergistically to provide the research community with a valued resource.

Development of PAZAR will require ongoing effort to expand the data represented, the means to access the data and the quality of the data curation tools. At present, existing data collections are being added to PAZAR with the permission and collaboration of the boutique operators. We anticipate the boutique database creators will be strongly motivated to use the system as it eases their own work. For instance, most high-throughput datasets currently generated never become available through a database and web interface because of the limited time researchers want to put into this effort. PAZAR provides an easy way to make these data available and to maintain them. Readers of this paper are encouraged to consider opening a boutique or working with the PAZAR team to move an existing data collection into the system.

Our goal is for PAZAR to become the public repository for data and annotations pertaining to transcriptional regulation. By promoting strong integration with tools for computational analysis and prediction of cis-regulatory sequences, boutique database operators will be motivated to participate in the expansion of the system.

## Abbreviations

API, application programming interface; CRE, cis-regulatory element; GFF, general feature format; TF, transcription factor; XML, extensible markup language.

## Authors' contributions

EPC and SK participate in creating the vision of the system, designed the database and implemented the software. EPC prepared the initial draft of the manuscript. JL participated in the software and database design. SL, MS and AT tested the system, developed documentation, and compiled the Pleiades data collection. AT produced the Cytoscape figure and contributed to the importation of OregAnno data. JS co-supervised the project and participated in the creation of the vision. WWW co-supervised the project, participated in the design of the system, and revised the manuscript. All authors read and provided feedback on the manuscript.
